# LanCL proteins are not Involved in Lanthionine Synthesis in Mammals

**DOI:** 10.1038/srep40980

**Published:** 2017-01-20

**Authors:** Chang He, Min Zeng, Debapriya Dutta, Tong Hee Koh, Jie Chen, Wilfred A. van der Donk

**Affiliations:** 1 Department of Chemistry and Howard Hughes Medical Institute, University of Illinois at Urbana-Champaign, Urbana, Illinois, United States of America; 2 Department of Cell and Developmental Biology, University of Illinois at Urbana-Champaign, Urbana, United States of America

## Abstract

LanC-like (LanCL) proteins are mammalian homologs of bacterial LanC enzymes, which catalyze the addition of the thiol of Cys to dehydrated Ser residues during the biosynthesis of lanthipeptides, a class of natural products formed by post-translational modification of precursor peptides. The functions of LanCL proteins are currently unclear. A recent proposal suggested that LanCL1 catalyzes the addition of the Cys of glutathione to protein- or peptide-bound dehydroalanine (Dha) to form lanthionine, analogous to the reaction catalyzed by LanC in bacteria. Lanthionine has been detected in human brain as the downstream metabolite lanthionine ketimine (LK), which has been shown to have neuroprotective effects. In this study, we tested the proposal that LanCL1 is involved in lanthionine biosynthesis by constructing LanCL1 knock-out mice and measuring LK concentrations in their brains using a mass spectrometric detection method developed for this purpose. To investigate whether other LanCL proteins (LanCL2/3) may confer a compensatory effect, triple knock-out (TKO) mice were also generated and tested. Very similar concentrations of LK (0.5–2.5 nmol/g tissue) were found in LanCL1 knock-out, TKO and wild type (WT) mouse brains, suggesting that LanCL proteins are not involved in lanthionine biosynthesis.

The biosynthesis of lanthipeptides, or lanthionine-containing peptides, has been investigated extensively^1^. In general, ribosomally synthesized precursor peptides undergo a series of post-translational modifications in which serines and threonines are dehydrated to dehydroalanine (Dha) and dehydrobutyrine (Dhb). The dehydrated amino acids form thioether crosslinks via reaction with the side chains of cysteine residues catalyzed by lanthionine cyclase (LanC). Homologs of LanCs exist in a wide variety of eukaryotic organisms, including animals and plants. These LanC-like (LanCL) proteins share conserved hydrophobic domains, zinc-binding residues^2^, and putative active site residues with bacterial LanC^3^. Three genes coding for the LanCL proteins are present in the human genome, with LanCL1, LanCL2 and LanCL3 encoded on chromosomes 2^4^, 7^5^ and X, respectively. LanCL1 and LanCL2 have been shown to be highly expressed in various parts of the brain and testis in human^4^,^6^,^7^ and mice^8^, along with lower expression in most other tissues examined^6^.

Since the discovery of LanCL proteins, much effort has focused on understanding their function(s). LanCL2 plays roles in regulating adriamycin sensitivity^9^,^10^ and abscisic acid signaling^11^,^12^,^13^,^14^, and the protein is also a novel regulator of Akt phosphorylation by binding to and enhancing the kinase activity of mTORC2^15^. LanCL1 appears to have different functions. LanCL1 was identified as a reduced glutathione (GSH)-binding protein in bovine brain^16^ and breast cancer cells^17^. Chung *et al*. showed the expression level of LanCL1 was increased during the onset of amyotrophic lateral sclerosis (ALS) in a mouse model (SOD1^G93A^), implicating a possible role of LanCL1 in this disease^16^. Most recently, Huang *et al*. reported an anti-oxidant function of LanCL1 in the central nervous system based on studies using LanCL1 knockout mice^8^. Paradoxically, Zhong *et al*. suggested that LanCL1 may have a negative effect on neuroprotection^18^. In this study, LanCL1 was reported to have an inhibitory effect on the activity of cystathionine-β-synthase (CβS), a key enzyme in transforming neurotoxic homocysteine to cysteine. Decrease in the cellular GSH/GSSG ratio disrupted the LanCL1-CβS interaction and increased the activity of CβS, which boosted the level of cysteine and thus GSH, therefore protecting cells from oxidative stress. However, the efficiency of GSH formation resulting from increased CβS activity has been questioned recently^19^.

Lanthionine has been detected in mammals as lanthionine ketimine (LK), formed via monodeamination by glutamine transaminase K and spontaneous cyclization^20^,^21^. To date, the physiological functions of lanthionine and LK have remained elusive. LK binds to the bovine brain membrane in a specific, reversible manner with high affinity (K_d_ = 58 nM), suggesting a neurotransmitter-like function^22^,^23^. Intriguingly, LanCL1 was shown to interact with LK in the mammalian brain^24^, and more recently, a cell-permeable ester derivative of LK (LKE) was shown to have anti-inflammatory and anti-apoptotic function in NSC-34 motor neuron-like cells^24^. A neuritogenesis effect of LKE was demonstrated in primary chick dorsal root ganglia culture, and LKE treatment delayed the onset of clinical paralysis and increased the survival of SOD1^G93A^ mutant mice^24^. Moreover, in a mouse model of Alzheimer’s disease, administration of LKE also substantially mitigated cognitive decline^25^, and LKE was found to protect neuronal cells in a mouse model of cerebral ischemia^26^. These *in vivo* results suggest neuroprotective and neuritogenic activity of LK.

Linking the relative abundance of LanCL1 in brain, the presence of LK in brain, and the observed interaction of LK and LanCL1, Hensley *et al*. proposed that lanthionine or LK could be an allosteric regulator of LanCL1 or the product of a LanCL1-catalyzed reaction^24^. Since LanCL1 binds to the Cys of glutathione^3^, and because bacterial LanC catalyzes addition of Cys to dehydrated Ser residues in peptides, it was suggested that glutathione could be one of the substrates of LanCL1 during lanthionine formation (Fig. 1)^24^. Indeed, precedent in Nature for amino acid crosslinking in a protein followed by proteolysis to generate a bioactive small molecule is found in the biosynthesis of thyroid hormone^27^.

In this study, we tested the hypothesis that LanCL1 may be involved in LK biosynthesis by creating a LanCL1 knockout (KO) mouse line and quantifying LK in the brain. Concentrations of about 0.5–2.5 nmol/g tissue were detected in both WT and LanCL1 KO mouse brains, suggesting LanCL1 may not play a major role in lanthionine synthesis *in vivo*. To rule out a potential compensatory effect from other LanCL proteins, triple knock-out (TKO) mice were also generated. The LK levels in WT and TKO mice were similar, confirming that despite the chemically appealing hypothesis, LanCL proteins are likely not involved in LK synthesis.

## Results

### Generation of Lancl1, Lancl2 and Lancl3 knock-out mice

Zinc finger nuclease (ZFN) mRNA designed to target the third exon of the LanCL1 gene was injected into FVB embryos at the pronuclear stage followed by transfer into pseudo pregnant female FVB mice, resulting in seventy three founder mice. Genotyping of tail DNAs by PCR-based Surveyor assay revealed that eight founder mice harbored mutations at the expected ZFN cleavage site. The PCR products were subcloned and sequenced to characterize the mutations. Notably, each PCR product contained a mixture of wild-type and mutant sequences, suggesting that all founder mice were mosaic. All eight genomic DNAs were confirmed to contain deletions of 1-nucleotide to 48-nucleotides at the expected ZFN site. We chose a founder mouse with a 19-nucleotide deletion (Fig. 2a) to breed with wild-type FVB mice for establishing a germline-transmitted mutant mouse line and further characterization. Heterozygous and homozygous mutant mice were identified by PCR genotyping (Supplementary Fig. S1a), and a Mendelian ratio was observed for both genotypes. The 19-nucleotide deletion in exon 3 would result in a frame shift in the remaining coding sequence, and the resulting premature stop codon in the mRNA would lead to nonsense-mediated mRNA decay and hence knockout of the gene product. Tissues were harvested from young adult mice, homogenized, and subjected to western blot analysis. Complete loss of the LanCL1 protein was confirmed in the brain, heart, and liver of homozygous mutant mice (Supplementary Fig. S1e). From here on, we refer to the homozygous mutant mice as Lancl1−/−.

Similar procedures were performed to generate Lancl2−/− and Lancl3−/− mice. The resulting LanCL2 KO mouse line bears a 2-nt deletion at the ZFN target site in exon four and the LanCL3 KO mouse line has a 37-nt deletion in exon one (Fig. 2a). The homozygous mutant mice were identified by genotyping (Supplementary Fig. S1b,c), and complete loss of the target proteins was confirmed by western blotting (Fig. 2b–d and Supplementary Fig. S1f). All Lancl−/− mice displayed no gross abnormality in phenotype. To check if any compensatory effects exist in the LanC-like protein family, the expression of all three LanCL proteins was examined in side-by-side experiments for all three single LanCL KO mouse lines. The results showed that the KO of any single LanCL protein did not affect the expression of the other two LanCL proteins (Fig. 2b–d).

### Generation and validation of LanCL triple knock-out (KO) mice

Double KO mouse lines were generated by crossing single KO mice. After three rounds of breeding, all three double KO mouse lines were obtained. We then crossed double KO mouse lines to generate LanCL1/2/3 triple KO mice. The complete knock-out of all three Lancl genes was verified by genotyping (Supplementary Fig. S1d). Brain tissue was collected, homogenized and subjected to western blotting. The absence of all three LanCL proteins confirmed the successful generation of the TKO mouse line (Fig. 2e). Again, no gross abnormality was observed in the TKO mice.

### PITC detection of LK in WT and LanCL1 KO mouse brains

Cavallini and colleagues reported a method for detection and quantification of LK, which involves reaction with phenylisothiocyanate (PITC) and subsequent analysis of the absorbance of the product (PTH-LK) at 380 nm^28^,^29^. Using this method, LK has been shown to be present in human and bovine brain at a concentration of 0.5–1 nmol/g tissue^30^,^31^, as well as in human urine samples with a relative concentration of 3–140 μg/g creatinine^28^. We initially used this method of LK detection. First, a synthetic standard was derivatized with PITC and the product analyzed by HPLC with detection at 380 nm to determine the elution time (Fig. 3a). Then WT mouse brain homogenate was treated with the derivatization reagents, and the resulting sample was analyzed under the same conditions (Fig. 3b). Standard synthetic LK was subsequently spiked into the mouse brain homogenates before sample preparation. The peak with a retention time of 25.8 min increased in intensity, indicating that it corresponds to LK (Fig. 3c). As a negative control, brain sample that was not treated with the derivatization reagents showed no corresponding peak. To further characterize the material giving rise to the peak, the fraction eluting at 25 min was subjected to liquid chromatography (LC) coupled to tandem mass spectrometry (MS/MS) analysis using multiple reaction monitoring (MRM), which confirmed that the peak corresponded to derivatized LK (Supplementary Fig. S2d). Using the optimized PITC method, brain LK levels in thirteen WT mice were determined as shown in Fig. 3d. We observed rather small HPLC absorbance values at 380 nm, indicating small amounts of LK in WT mouse brains.

### LC/MS/MS detection of lanthionine metabolites in WT and LanCL1 KO mice

Because the detected amounts of PTH-LK in WT mouse brain approached the detection limit of HPLC-UV (Fig. 3b), we investigated mass spectrometry based detection without derivatization. To detect LK with higher sensitivity and specificity, a triple quadrupole mass spectrometer coupled with HPLC was employed using MRM looking at specific mass transitions in tandem electrospray ionization MS. Unlike the PITC method, which only detected LK, MS enabled the detection of all related metabolites. To obtain a more comprehensive assessment of the *in vivo* lanthionine level, specific methods were developed to directly detect lanthionine and LK. The possible sodium adduct of LK (LK-Na) was also examined (Supplementary Fig. S2), and a specific fragmentation transition of 190.1 Da → 144.1 Da was chosen to detect and quantify LK. Figure 4a shows a typical MRM chromatogram of WT mouse brain, illustrating that the signal-to-noise of LK detection in WT mouse brain using this method was high. Free lanthionine and LK-Na were also observed in mouse brain sample but with much lower signal intensity compared to standards (Supplementary Fig. S2b,c), suggesting they are a minor contribution to lanthionine species. Because of the higher levels of LK and much better resolution, we focused on quantifying the LK levels in mouse brain.

### Quantification of LK levels in mouse brain with isotopically-labeled LK

The LC/MS/MS method provided more straightforward detection of LK and related metabolites than the PTH-LK method and is less likely to be affected by other metabolites because no derivatization is needed. To further improve quantification, an isotopically labeled synthetic internal standard was employed. The synthetic isotope-labeled LK was prepared from uniformly ^13^C- and ^15^N-labeled Cys and has a 4 Da mass increase compared to unlabeled LK. After optimization of conditions, a transition of 190.1 Da → 144.1 Da was chosen for LK and 194.1 Da → 148.0 Da for isotopically labeled LK. Both endogenous LK and labeled LK eluted at 7.4 min in samples from WT and LanCL1 KO mouse brains (Fig. 4). Mouse brain to which isotopically-labeled LK was not added served as a negative control; no signal was observed for the 194.1 Da → 148.0 Da transition indicating that endogenous metabolites do not coincidentally fragment to give the same transition. Next, we spiked in known concentrations of isotope-labeled LK and measured the concentrations using the indicated transition with synthetic standards to generate a standard curve. Endogenous LK concentrations were calculated by comparing peak areas with isotope labeled LK of known concentration added to the brain samples. In order to achieve a higher intensity of peak signal, two or three mouse brains were pooled. The same method was applied to TKO mouse brains. We detected LK concentrations in brains of four, two and one month old mice and the results are summarized in Fig. 5. Compared with bovine brain, mouse brain has a slightly higher concentration of LK at 0.5–2.5 nmol/g tissue. More importantly, no statistically significant difference was observed between WT, LanCL1 KO, and TKO mouse brains of all ages examined.

## Discussion

The biogenesis of lanthionine in mammals is elusive. Lanthionine has long been thought to be a by-product from the transsulfuration pathway. The pyridoxal phosphate (PLP)-dependent enzyme cystathionine β-synthase (CβS) catalyzes the first step in the transsulfuration pathway, condensing homocysteine with serine or cysteine to form cystathionine, a bis-amino acid detected in the same organs as lanthionine^28^,^31^. Cystathionine γ-lyase (CSE) catalyzes the second step in the pathway, the α,γ-elimination of cystathionine to give cysteine, α-ketobutyrate and ammonia (Supplementary Fig. S3a). It was reported that CβS and CSE can also catalyze condensation of cysteine with either serine or another cysteine to form lanthionine (Supplementary Fig. S3b). This activity was observed *in vitro* as a minor reaction^32^,^33^, and evidence of CβS or CSE being the biosynthetic enzymes for lanthionine *in vivo* is missing. The discovery of mammalian homologs of bacterial lanthionine forming enzymes led Hensley and colleagues to propose that they may be involved in lanthionine formation in mammalian cells^24^. LanCL1 was a particularly attractive target for lanthionine formation in brain because it binds LK, binds glutathione, is ubiquitously present in brain, and appears to perform an antioxidative, neuroprotective function in brain, similar to the activities displayed by LKE.

**Figure 1:**
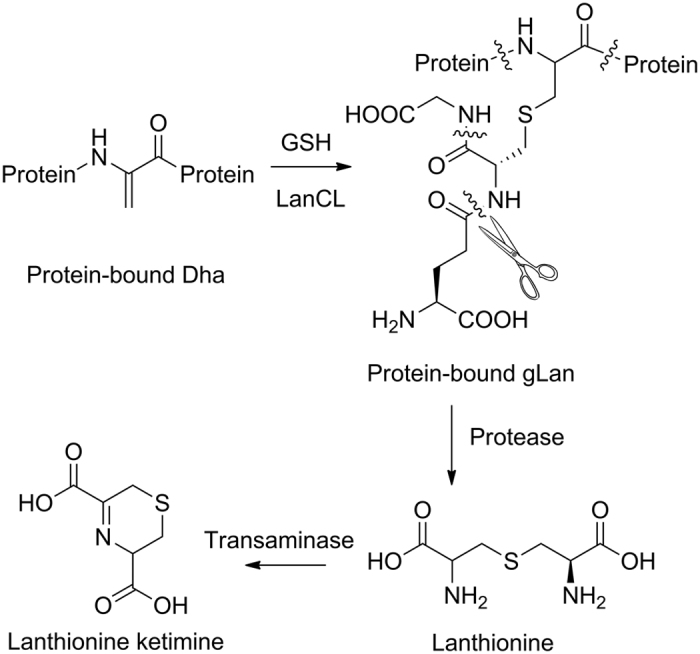
Proposed pathway of lanthionine synthesis by LanCL1^24^. LanCL1 may catalyze the conjugation of GSH to protein-bound Dha, followed by peptidase-mediated digestion of the reaction product (proteolysis sites indicated by curled lines) to form lanthionine. Transamination to form lanthionine ketimine has been documented previously. An enamine tautomer can also be formed (not shown).

**Figure 2:**
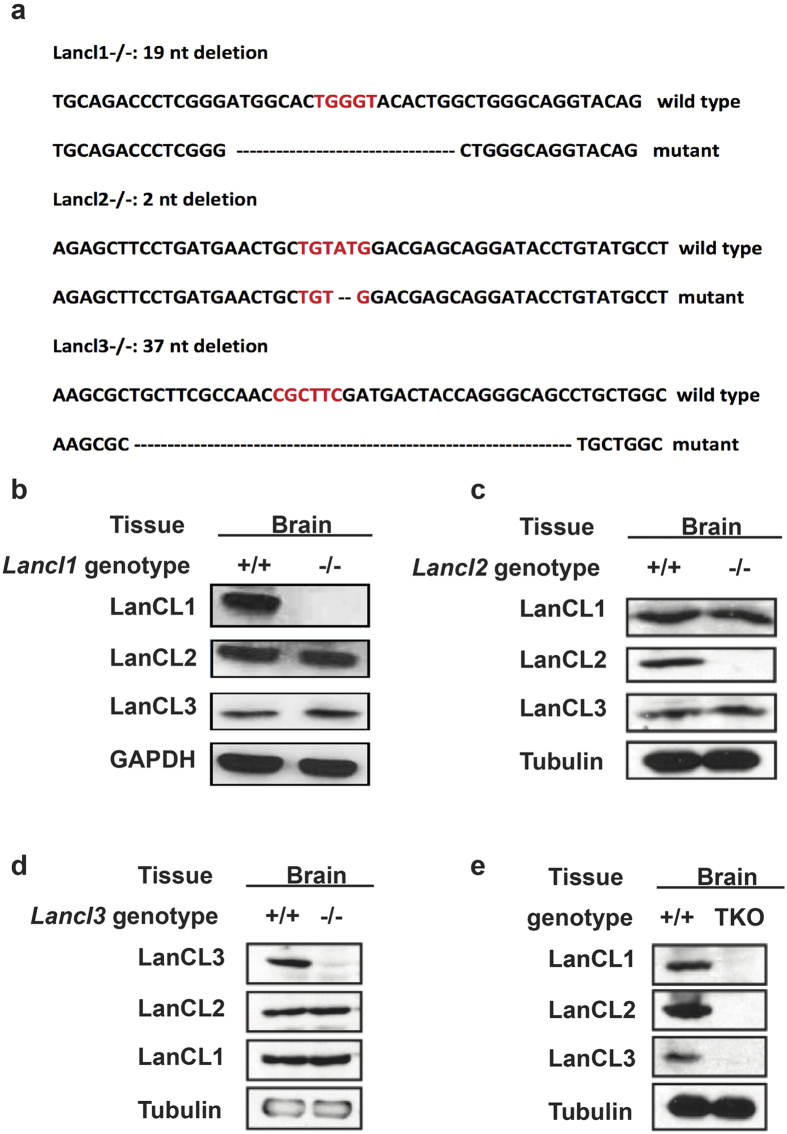
LanCL protein expression in different LanCL KO mouse lines. (**a**) Genomic sequences indicating the mutations in Lancl1−/− (#36), Lancl2−/− (#52) and Lancl3−/− (#67) mice. Red letters indicate ZFN cutting site. Deleted nucleotides in mutant mice are shown as dashes. (**b–e**) Western blotting confirmed the complete KO of LanCL proteins in the corresponding LanCL KO mouse lines. (**b**) Lancl1−/−, (**c**) Lancl2−/−, (**d**) Lancl3−/− and (**e**) TKO mice were sacrificed with age- and gender-matched WT mice. Protein was extracted from brains and subjected to Western blotting with loading control.

**Figure 3:**
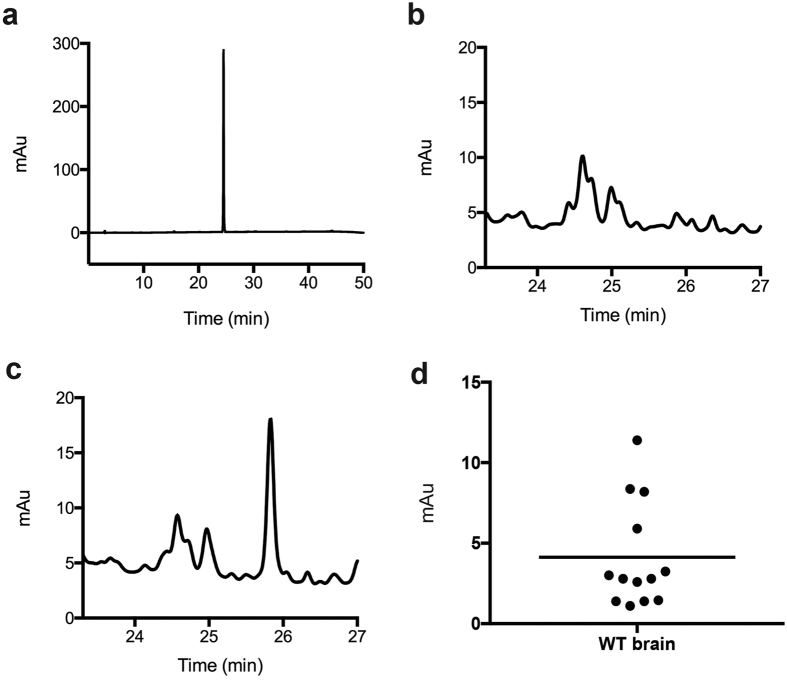
HPLC analysis of PTH-LK. (**a**) LK standard after PITC derivatization (PTH-LK). (**b**) WT mouse brain homogenate after PITC derivatization. (**c**) WT mouse brain homogenate spiked with synthetic LK showed an increased PTH-LK signal after PITC derivatization. (**d**) LK level in WT mouse brains. Each data point represents one mouse. The mean value is shown.

**Figure 4:**
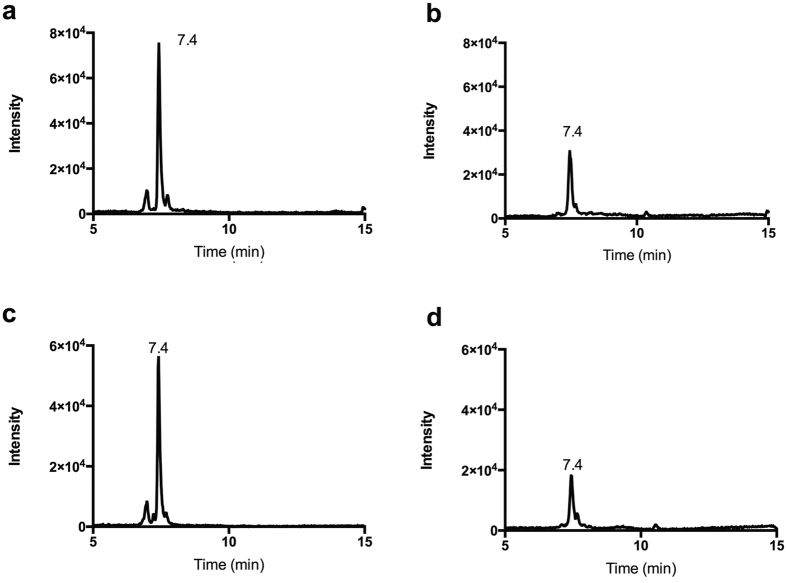
Multiple reaction monitoring (MRM) of LK. MRM chromatogram for the transition of (**a**) 190.1 → 144.1 Da for LK, and (**b**) 194.1 → 148.0 Da for isotopically labeled LK in WT mouse brain. Also shown are the corresponding transitions (**c**) 190.1 → 144.1 Da and (**d**) 194.1 → 148.0 Da in LanCL1 KO mouse brain. The elution time for each peak is shown.

**Figure 5:**
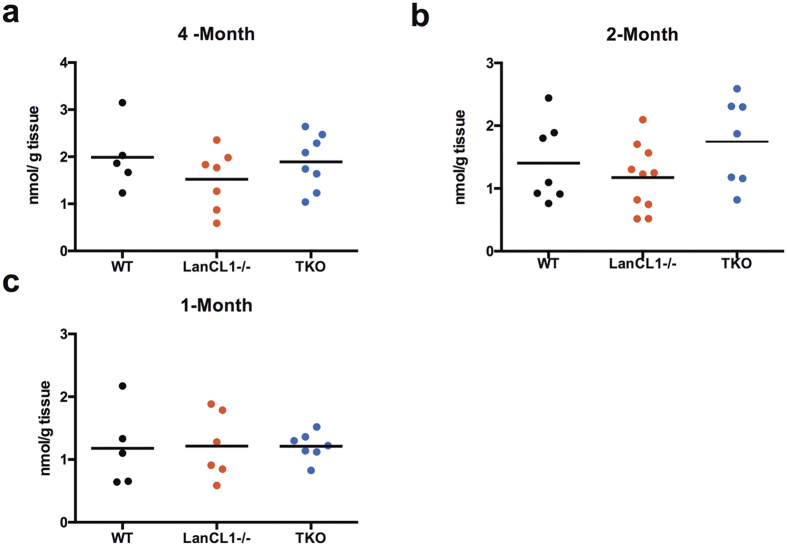
LC/MS/MS quantification of LK level in WT, LanCL1 KO and TKO mouse brains. Data obtained from (**a**) four-month, (**b**) two-month and (**c**) one-month old mice. Each data point represents the average level of 2–3 mouse brains. No significant difference was observed (P > 0.05).

A seemingly missing piece in this hypothesis is the mechanism by which the Dha would be formed. The bacterial lanthipeptide enzymes that dehydrate Ser to Dha do not have mammalian sequence homologs, but alternative routes of generating Dha have been reported^34^. Therefore, Hensley and coworkers proposed that LanCL1 may catalyze the addition of the Cys in glutathione to a protein- or peptide-bound Dha, followed by trimming of the product by peptidases to form lanthionine (Fig. 1). In support of the model, the adduct of GSH to dehydroalanine (glutathionyl lanthionine, gLan) was synthesized and shown to have biological effects similar to that of LK in ALS mouse models^24^. Intriguingly, a very recent crystal structure of one of the bacterial enzymes that dehydrates Ser shows that structurally they strongly resemble mammalian lipid kinase-like proteins^35^, suggesting that perhaps enzymatic formation of Dha in mammals is indeed feasible. We therefore set out to test the involvement of LanCL1 in lanthionine formation.

LK has been previously detected in human and bovine brain by PITC derivatization and subsequent HPLC analysis relying on the absorbance of the PTH-LK derivative^22^,^23^. Here we report the detection of LK in mouse brain using both PITC derivatization and a new LC/MS/MS method. A peak corresponding to PTH-LK was detected in WT and LanCL1 KO mouse brain with low intensity that approached our detection limit. Therefore, an LC/MS/MS method was developed in which LK was directly detected without a derivatization step. Use of isotope labeled LK as internal standard, which is expected to undergo the same potential losses during sample preparations, was employed to further reduce quantification error. The concentration of LK in mouse brain determined by the LC/MS/MS method was 0.5–2.5 nmol/g tissue, which is somewhat higher than that found in bovine brain by the PITC method. The LK levels in four, two and one month old mouse brains showed no significant differences, indicating the production of LK is not strongly age dependent.

LK was detected in both WT and LanCL1 KO mouse brains by both methods at similar concentrations, suggesting that LanCL1 is not required for LK biogenesis. Furthermore, the similar concentrations of LK in TKO mouse brains ruled out a compensatory effect and more importantly, suggested that the three LanCL proteins are not involved in LK synthesis *in vivo*. Whether LK found in mammals is a by-product of CβS or CSE or whether there exists another biosynthetic route is currently not clear. We can also not rule out that LK is derived from commensal bacteria, many of which produce lanthipeptides. We note that our results do not exclude the possibility of other types of functional correlation between LanCL1 and LK, such as the aforementioned allosteric effector model. A systematic screening for potential LanCL1 co-substrates is needed to define its function in neuroprotection.

## Methods

### Antibodies and Chemicals

Anti-LanCL1 antibody was purchased from Bethyl Laboratories (Montgomery, TX). Anti-LanCL2 antibody was generated by Proteintech Group (Chicago, IL) using full length recombinant LanCL2 as the antigen as previously described^15^. Anti-LanCL3 antibody was from Sigma-Aldrich (St. Louis, MO). Stable-isotope labeled cysteine (^13^C_3_, ^15^N) and DMSO-d_6_ were purchased from Cambridge Isotope Laboratories (Andover, MA). PITC was purchased from Perkin Elmer (Waltham, MA). Triethylamine and pyridine were purchased from Fisher Scientific (Waltham, MA), and acetonitrile from Macron Fine Chemicals (Center Valley, PA). Other chemicals were from Sigma Aldrich (St Louis, MO).

### Synthesis of LK standard

LK was synthesized as described by Ricci *et al*.^36^ with minor modifications. L-Cysteine (2 mmol) was suspended in 0.4 mL of 5 M HCl and fully dissolved by addition of 1.2 mL of H_2_O. To the resulting solution was added 2 mmol of bromopyruvic acid in 0.6 mL of H_2_O. A light yellow solid formed after gentle swirling for 5 min. The solid was filtered and washed with cold water and dried over P_2_O_5_. The identity of the product was verified by NMR spectroscopy.

### PITC derivatization

Mouse brain sample preparation and PITC derivatization were performed essentially as described previously with minor modifications^28^. Freshly harvested mouse brain (about 0.5 g of either WT or KO) was homogenized four times with an OMNI TH homogenizer in 5 mL of 30% acetonitrile/water using 30 s pulse and pause cycles. The homogenate was deproteinated by adjusting the acetonitrile/water ratio to 2:1 and centrifuged at 23,700 × *g* for 10 min. The supernatant was concentrated under N_2_ flow to 1 mL before PITC derivatization. One mL of concentrated brain homogenate was mixed with 90 μL of PITC and 3 mL of coupling buffer (acetonitrile:pyridine: triethylamine:H_2_O = 10:5:2:3) in a 5 mL reaction vial and stirred for 30 min at 20 °C. The solution was then dried at 40 °C using a rotary evaporator and re-dissolved in 1 mL of 10 mM potassium acetate buffer (pH = 8.0). Standard LK (100 ng) was dissolved in 30% acetonitrile/water, followed by addition of 1 mL of coupling buffer and 30 μL of PITC. Reaction conditions and work-up were identical to those described for the brain sample.

### Mouse husbandry and microinjection

All animal experiments in this study followed protocols approved by the Animal Care and Use Committee at the University of Illinois at Urbana-Champaign. FVB mice were maintained on a 12 h/12 h light/dark cycle with access to water and food. Microinjections of ZFN mRNAs (Sigma) were performed in the Transgenic Mouse Facility in the Roy J. Carver Biotechnology Center at the University of Illinois at Urbana-Champaign. LanCL1, LanCL2, and LanCL3 ZFN mRNAs were diluted to 2.5–4 ng/μL with 10 mM Tris buffer, 0.1 mM EDTA (pH 7.5) and injected in FVB embryos at the pronuclear stage before transferring to pseudo pregnant females.

### Genomic DNA isolation from mouse tail tips

For PCR amplification followed by Surveyor mismatch endonuclease assay^37^, mouse tail tips (2–5 mm) were dissolved by incubating with 600 μL of extraction buffer (20 mM Tris pH 7.5–8.0, 50 mM EDTA, 100 mM NaCl, 0.5% SDS and 500 μg/mL proteinase K) for 2–3 h at 55 °C. Then 240 μL of high salt solution (4.21 M NaCl, 0.63 M KCl, 10 mM Tris pH 8.0) was added to the dissolved tail and the sample was incubated for 30 min at 4 °C to precipitate proteins. The precipitated proteins were removed by centrifugation at 16,100 × *g* for 10 min at 4 °C and the supernatant was transferred to a new tube. Genomic DNA was precipitated by adding 2x volume of ethanol followed by centrifugation at 16,100 × *g* for 10 min at 4 °C. The precipitated DNA pellet was washed once with 80% ethanol and dissolved in 200–400 μL of TE buffer (1 mM Tris pH 8.0, 0.1 mM EDTA) after a brief air dry. For PCR amplification followed by agarose gel examination, genomic DNA was extracted from mouse tail tips using KAPA Express Extract Kits following the manufacturer’s instructions.

### PCR for Surveyor assays

The following primers were used for PCR. LanCL1ZFN_F: 5′ TCC ATA TGT GGT TTC TGA AAA GC 3′; LanCL1ZFN_R: 5′ AGC GCC AGG CAT GAA TAC 3′; LanCL2ZFN_F: 5′ CAA AGC TGG AGA TTC AAT TTA GG 3′; LanCL2ZFN_R: 5′ AAG CAG AGG CTG GGT GAT AA 3′; LanCL3ZFN_F: 5′ GTC TTG TCA CCT CCC GTC TC 3′; LanCL3ZFN_R: 5′ GCT CTG GGA GAC GTG GTA GA 3′. The annealing Tm was 55 °C, 58 °C and 56 °C for LanCL1, LanCL2 and LanCL3 PCR, respectively. The following PCR program was used for PCR for Surveyor assay and genotyping PCR with different annealing temperatures: 94 °C for 2 min, 30 cycles of 94 °C for 30 s, 58 °C/55 °C/56 °C (for LanCL1/LanCL2/LanCL3) for 1 min, 72 °C for 30 s, and 72 °C for 10 min.

### Founder identification using Surveyor mismatch endonuclease assay

Genomic DNA was extracted from mouse tail tips cut from three week old pups and a 400 bp fragment surrounding the ZFN cutting site was amplified by primer sets ZFN_F and ZFN_R. The PCR product (≥50 ng/μL) was denatured and hybridized using the following program: 95 °C for 10 min; 95 °C to 85 °C, −2 °C/s; 85 °C to 25 °C, −0.1 °C/s; 4 °C, indefinitely. The rehybridized PCR product (about 15 μL) was incubated with 1 μL of enhancer S and 1 μL of Nuclease S (Integrated DNA Technologies) for 1 h at 42 °C. The cleaved products were resolved by 3% NuSieve DNA agarose gel electrophoresis.

### PCR Genotyping of Lancl1−/−, Lancl2−/−, and Lancl3−/− mice

For Lancl1 and Lancl3, the same PCR was performed as for the Surveyor assay. For Lancl2, two pairs of primers were designed to genotype the 2-nt deletion mutant: 2 ntWT, CTT CCT GAT GAA CTG CTG TAT; 2 ntMUT, TTC CTG ATG AAC TGC TGT GG; 200 bpREV1, GAA TTA CTC CCA CCT AGA AGC; 400 bpREV1, GCT CTA TTT GGT TAT GTG GGT. Primers 2 ntWT and 200 bpREV1 were paired to amplify a 200 bp DNA fragment around the ZFN cutting site only from WT mice, whereas primers 2 ntMUT and 400 bpREV1 would amplify a 400 bp DNA fragment around the ZFN cutting site only from the 2 nt deletion mutant. PCR products were resolved by 1.5% agarose gel electrophoresis. The mouse lines have been deposited at the Jackson Laboratory (029694 for LanCL1−/− and 029695 for LanCL2 −/−).

### RNA and protein extraction from mouse tissues

Mouse brain, heart and liver were dissected and snap frozen immediately in liquid nitrogen. Frozen tissues were homogenized by grinding with a pestle and mortal. For protein extraction, the homogenized powder was lysed in tissue extraction buffer (200 nM Tris HCl pH 7.4, 150 mM NaCl, 1 mM EGTA, 1 mM EDTA, 2.5 mM sodium pyrophosphate, 1 mM β-glycerol phosphate, 1 mM sodium orthovanadate, 2% Triton X-100 and a protease inhibitor cocktail) and rotated for 30 min at 4 °C. After centrifugation at 16,100 × *g* for 10 min, the supernatant was transferred to a new tube and boiled with 1x volume of 2x Laemmili sample buffer for SDS-PAGE and Western blotting. RNA was extracted from the homogenized powder using the RNeasy Kit (QIAGEN) following the manufacturer’s instructions.

### LC/MS/MS of mouse brain samples

Freshly harvested mouse brain (about 0.5 g of either WT or KO) was homogenized four times with an OMNI TH homogenizer in 5 mL of 30% acetonitrile/water using 30 s pulse and pause cycles. Homogenate was separated into two aliquots. To one aliquot, isotopically labeled LK standard (100 ng) was added. The other aliquot was processed without addition of labeled LK. The two aliquots were deproteinated by addition of acetonitrile to adjust the acetonitrile/water ratio to 2:1 and centrifuged at 23,700 × g for 10 min. The supernatant was collected and dried under N_2_ flow. Dried sample was resuspended in 400 μL of 30% acetonitrile/water followed by centrifugation at 16,100 × g for 5 min. The supernatants were then collected for LC/MS/MS. Samples were analyzed with a 5500 QTRAP LC/MS/MS system (AB Sciex, Foster City, CA) in the Metabolomics Laboratory of the Roy J. Carver Biotechnology Center, University of Illinois at Urbana-Champaign. The 1200 series HPLC system (Agilent Technologies, Santa Clara, CA) includes a degasser, an autosampler, and a binary pump. The LC separation was performed on an Agilent Zorbax SB-Aq column (4.6 × 50 mm, 3.5 μm), with a gradient from 100% A (0.1% formic acid in water) to 99% B (0.1% formic acid in acetonitrile) in 6 min at a flow rate of 0.45 mL/min. The autosampler was set at 5 °C. The injection volume was 5 μL. Positive and negative ion mass spectra were acquired under electrospray ionization (ESI) with the ion spray voltage at 5500 V and −4500 V, respectively. The source temperature was 500 °C. The curtain gas, ion source gas 1, and ion source gas 2 were 33 psi, 65 psi, and 50 psi, respectively. Multiple reaction monitoring (MRM) was used to measure related metabolites.

### HPLC

Analytical reversed-phase high-performance liquid chromatography (RP-HPLC) was performed on an Agilent 1260 Infinity System with a Hypersil Gold C18 column (250 mm × 4.6 mm, particle size 5 μ). The program ran from 98% buffer A (0.05 M ammonium acetate, pH 6.5) to 60% buffer B (acetonitrile: H_2_O = 7:3) in 30 min and then to 100% buffer B in 5 min at a flow rate of 1 mL/min. All HPLC solvents were filtered with a Millipore filtration system equipped with a 0.22 μm PVDF membrane filter prior to use.

### Statistical analysis

All data analysis was conducted using GraphPad Prism software V6.0 (GraphPad, San Diego, CA, USA). Comparisons of WT, LanCL1KO and TKO LK levels were made using one-way analysis of variance (ANOVA), assuming Gaussian distribution. Multiple comparisons were also conducted in conjunction with ANOVA by comparing the mean of one group with other two groups using Turkey’s test.

## Additional Information

**How to cite this article:** He, C. *et al*. LanCL proteins are not Involved in Lanthionine Synthesis in Mammals. *Sci. Rep.* **7**, 40980; doi: 10.1038/srep40980 (2017).

**Publisher's note:** Springer Nature remains neutral with regard to jurisdictional claims in published maps and institutional affiliations.

## Supplementary Material

Supplementary Information
