# Supplementary material for: LanCL proteins are not Involved in Lanthionine Synthesis in Mammals
Source: Sci Rep. 2017 Jan 20;7:40980. doi: 10.1038/srep40980 (PMC5247676; doi:10.1038/srep40980)
Supplement: Supplementary Information [file srep40980-s1.pdf]

SUPPLEMENTARY INFORMATION

LanCL proteins are not involved in lanthionine synthesis in mammals

Chang He[1], Min Zeng[2], Debapriya Dutta[1], Tong Hee Koh[1],

Jie Chen[2]*, and Wilfred A. van der Donk[1]*

[1]Department of Chemistry and Howard Hughes Medical Institute, University of Illinois at Urbana-Champaign, Urbana, Illinois, United States of America
[2]Department of Cell and Developmental Biology, University of Illinois at Urbana-Champaign, Urbana, United States of America

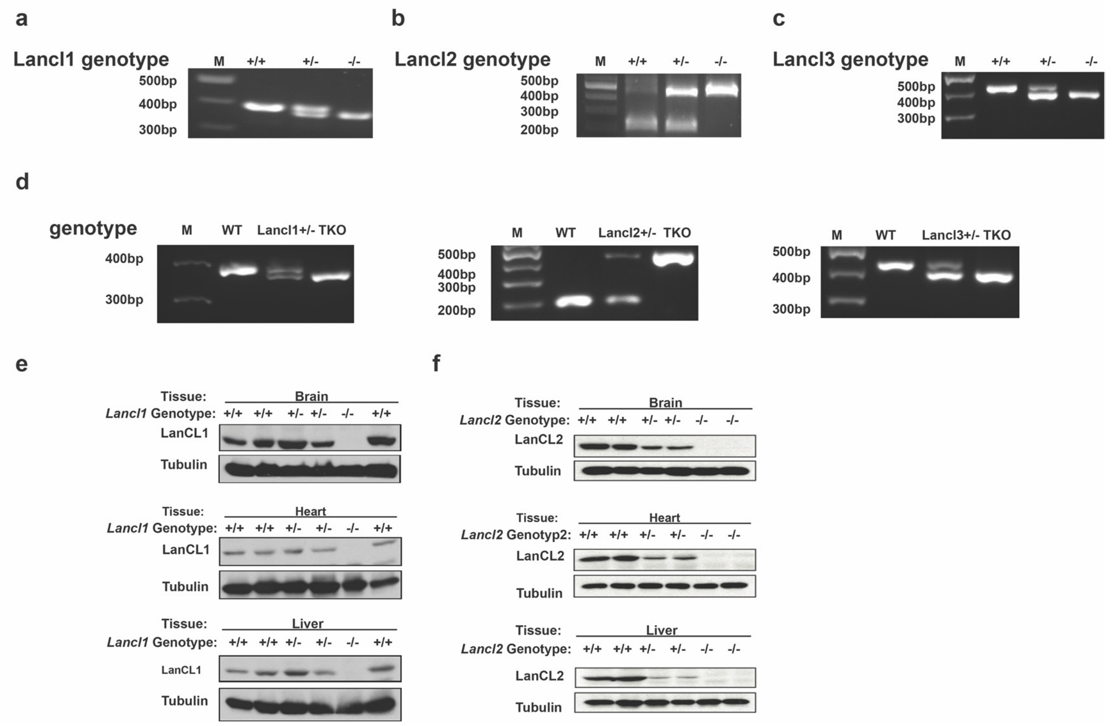

Supplementary Figure S1. LanCL1 KO mice genotyping and KO confirmation. (a-c) Genotyping results of single KO mice. Genotyping of **(a)** Lancl1-/-, **(b)** Lancl2-/- and **(c)** Lancl3-/- confirmed the generation of the corresponding single KO mice. For visualization purposes, a mixture of Lancl-/- and WT extracted DNA was used as Lancl+/- in panels a-c. **(d)** genotyping of TKO mice showed the complete deletion of all three Lancl genes. **(e-f)** Protein was extracted from brain, heart and liver tissue of Lancl1-/-, Lancl2-/- and wild type mouse with matched age and gender and subjected to western blotting. The complete deletion of LanCL proteins is seen in homozygous mice tissues.

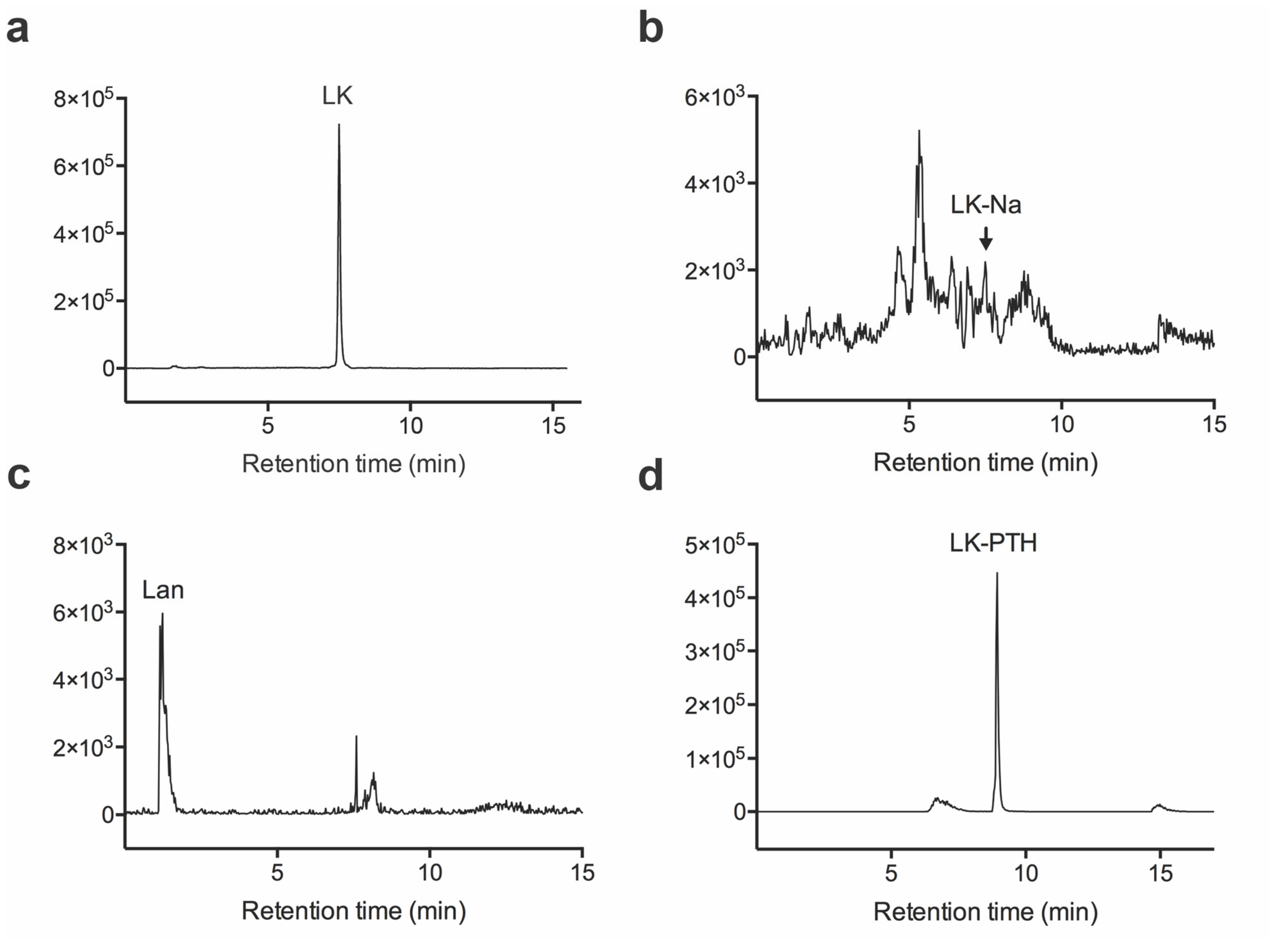

Supplementary Figure S2. MRM chromatograms of LK and related metabolites in WT mouse brain. WT mouse brain was homogenized and deproteinated. The supernatant was completely dried under N_2 flow and dissolved in 400 µl of 30% acetonitrile. An aliquot of 5 µL was injected for LC/MS/MS analysis of LK, LK-Na and Lan. LK-PTH was from the HPLC fraction corresponding to the PITC derivatized endogenous LK in WT mouse brain. **(a)** MRM chromatogram of detected LK (transition of 190.1 →73.1). **(b)** MRM chromatogram of detected LK-Na (transition of 212.0 → 168.1). **(c)** MRM chromatogram of detected Lan (transition of 202.0 → 120.0). **(d)** MRM chromatogram of detected LK-PTH (transition of 307.1 →126.1).

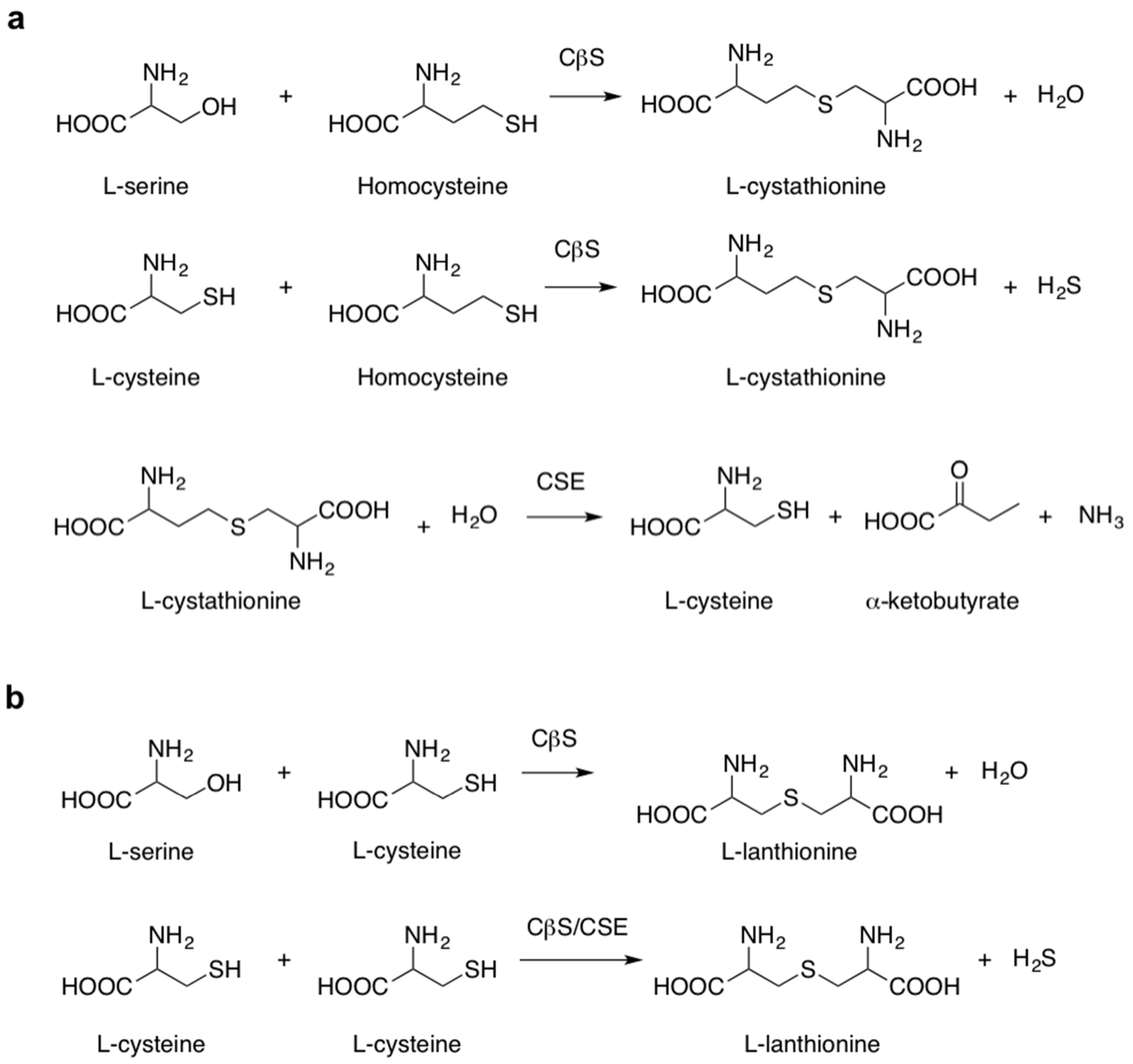

Supplementary Figure S3. Lanthionine formation through alternative reactions in the transsulfuration pathway. (a) The classic transsulfuration pathway catalyzed by CβS and CSE. **(b)** Alternative reactions catalyzed by CβS or CSE that lead to lanthionine formation. When the substrates are two molecules of cysteine, H_2S is formed instead of H_2O.
